# Supplementary material for: The genome sequence of the Shaggy Mining Bee, Lasioglossum villosulum (Kirby, 1802) (Hymenoptera: Halictidae)
Source: Wellcome Open Res. 2025 Oct 13;10:574. [Version 1] doi: 10.12688/wellcomeopenres.24870.1 (PMC12754356; doi:10.12688/wellcomeopenres.24870.1)
Supplement: Peer review history

# Reviewer report: Reviewer response for version 1

**DOI:** 10.21956/wellcomeopenres.27394.r140037

**Published:** 2026-01-07

**Authors:** Eduardo Zattara

**Affiliations:** 1 Instituto de Investigaciones en Biodiversidad y Medioambiente, Río Negro, Argentina

This data note reports the sequencing and assembly of the genome of a solitary species of sweat bee. Besides providing an instrumental resource for research on this particular species, it will also be informative when compared with genomes of other hymenopterans about the genomic evolutionary correlates of traits like sociality, size, behaviour, food preferences, etc. The report includes adequate detail both regarding the methodologies for sequencing and assembly, and quality assessment.

Are sufficient details of methods and materials provided to allow replication by others?

Yes

Is the rationale for creating the dataset(s) clearly described?

Yes

Are the datasets clearly presented in a useable and accessible format?

Yes

Are the protocols appropriate and is the work technically sound?

Yes

Reviewer Expertise:

Genomics, evolution of development, pollination biology

I confirm that I have read this submission and believe that I have an appropriate level of expertise to confirm that it is of an acceptable scientific standard.

# Reviewer report: Reviewer response for version 1

**DOI:** 10.21956/wellcomeopenres.27394.r140029

**Published:** 2025-12-30

**Authors:** Pilar De la Rúa

**Affiliations:** 1 University of Murcia, Murcia, Spain

The authors present the research flow for obtaining the genome of Lasioglossum villosulum. It appears that they have achieved a fairly complete version according to the data presented (N50 and single-copy genes present according to my search). It seems that these articles use a common template that always raises similar questions for me:

- The photo is not very good and the morphology of the specimen studied cannot be seen properly.
- The small dissected sample refers to a leg?
- On page 10 the scientific name of the specimen must be written in italics

Are sufficient details of methods and materials provided to allow replication by others?

Yes

Is the rationale for creating the dataset(s) clearly described?

Yes

Are the datasets clearly presented in a useable and accessible format?

Yes

Are the protocols appropriate and is the work technically sound?

Yes

Reviewer Expertise:

Conservation genetics

I confirm that I have read this submission and believe that I have an appropriate level of expertise to confirm that it is of an acceptable scientific standard.

# Reviewer report: Reviewer response for version 1

**DOI:** 10.21956/wellcomeopenres.27394.r136801

**Published:** 2025-11-13

**Authors:** Christopher Wyatt

**Affiliations:** 1 University College London, London, UK

The authors present the genome of *Lasioglossum villosulum, *which is of likely near complete genome based on the N50 and busco single copy genes present.

 The paper is clearly written and explained. I could not find any section that was unclear or needed correction. There is an appropriate background to the species with highly detailed methodology.

Are sufficient details of methods and materials provided to allow replication by others?

Yes

Is the rationale for creating the dataset(s) clearly described?

Yes

Are the datasets clearly presented in a useable and accessible format?

Yes

Are the protocols appropriate and is the work technically sound?

Yes

Reviewer Expertise:

Genomics, Evolutionary Biology, Bioinformatics

I confirm that I have read this submission and believe that I have an appropriate level of expertise to confirm that it is of an acceptable scientific standard.
